# An exploratory study on the impact of daily activities on the pleasure and physical activity of older adults

**DOI:** 10.1186/s11556-016-0170-2

**Published:** 2017-01-06

**Authors:** Miriam Cabrita, Richel Lousberg, Monique Tabak, Hermie J. Hermens, Miriam M.R. Vollenbroek-Hutten

**Affiliations:** 1 Telemedicine group, Roessingh Research and Development, P.O. Box 310, Enschede, 7522 AH The Netherlands; 2 Telemedicine group, Faculty of Electrical Engineering, Mathematics and Computer Science, University of Twente, P.O. Box 217, Enschede, 7500 AE The Netherlands; 3 Department of Psychiatry & Psychology, Maastricht University, Maastricht, The Netherlands

**Keywords:** Experience sampling method, Wearables, Positive emotions, Independent living, Active ageing

## Abstract

**Background:**

Pleasure is one determinant of intrinsic motivation and yet a dimension often forgotten when promoting physical activity among the older population. In this study we investigate the relation between daily activities and physical activity, experience of pleasure, and the interaction between pleasure and physical activity in the daily lives of community-dwelling older adults.

**Methods:**

Participants carried a hip-worn accelerometer during 30 consecutive days resulting in a total of 320 days of data collection. Current activity, location, companion and experience of pleasure during each activity were assessed through experience sampling on a smartphone every 1–2 h. Between- and within-individual differences were analysed with multi-level models and 10xN = 1 regression analysis.

**Results:**

Outdoor activities were associated with higher physical activity than indoor activities (*p* < 0.001). Performing leisure activities, outdoors and not alone significantly predicted pleasure in daily life (all p’s < 0.05). Being more active while performing leisure activities resulted in higher experiences of pleasure (*p* < 0.001). However, when performing basic activities of daily living (e.g. commuting or households) this relation was inverted. Results provide meaningful indication for individual variance. The 30 days of data collected from each participant allow for identification of individual differences.

**Conclusions:**

Daily activities and their contexts do influence the experience of pleasure and physical activity of older adults in daily life of older adults, although similar research with larger population is recommended. Results are in accordance with the literature, indicating that the method adopted (accelerometry combined with experience sampling) provides reliable representation of daily life. Identification of individual differences can eventually be automatically performed through data mining techniques. Further research could look at innovative approaches to promote Active Ageing using mobile technology in the daily life, by promoting physical activity through recommendation of pleasurable activities, and thus likely to increase the intrinsic motivation to become physically active.

## Background

An active lifestyle provides powerful benefits in the general health and wellbeing of the older adults aged 65 years and above. An adequate level of physical activity, combining aerobic activity, muscle-strength and balance training, improves the overall physical function, delaying functional decline and supporting independent living. To be physically active includes, but it is not limited to, participate in structured physical exercise. It also means to be active throughout the day, for example, by avoiding long periods of inactivity. Low-intensity walking activity is also suggested to be associated with better health [1]. The ‘Global Recommendations on Physical Activity for Health’ from the World Health Organization, highlight the importance of daily activities, such as household chores, games and transportation [2]. At the same time, older adults should maintain an active lifestyle also in terms of being engaged with their community and environment [3]. Some older adults can achieve an active lifestyle by themselves; however, others might benefit from an external nudge to become active [4]. Technology can play an important role here, by incorporating several strategies to support people in being physically active and actively engaged in their social environments.

The research presented in this manuscript is the first step towards the development of a tailored approach to promote physical activity and mental wellbeing in the daily lives of the older adults, through the recommendation of pleasurable, or enjoyable, activities. However, before designing an intervention, it is important to deepen the current knowledge on the interactions between physical activity, positive emotions and daily activities, being this the focus of the presented study. There are several reasons for this. First, participation in pleasurable activities in older age is associated with better physiological function and better sleep [5], improving general health and wellbeing. Second, there is growing evidence supporting the link between positive emotions and general health [6–10]. Third, according to the Broaden-and-Build Theory, those who experience higher levels of positive emotions are more likely to build a variety of resources, such as environmental mastery and social support [11]. These resources improve resilience to change, a very important characteristic for this population, as biological and social changes are likely to occur. Furthermore, the ‘upward spiral of lifestyle changes’ [12], a derivation from the previous theory, defends that positive emotions act as openers for acceptance and adoption of new behaviours, key characteristics when promoting behaviour change. Finally, according to the Self-Determination Theory, the enjoyment or pleasure experienced is an intrinsic motivator to repeat a certain activity [13], also already evaluated in the physical activity context [14]. One can thus hypothesize that people are more inclined to do what they like to do. This means that, when suggested an activity that they are familiar with, and have previous positive experiences with, they are more likely to follow the recommendation.

When aiming at increasing physical activity through promotion of pleasurable activity, it is important to take a deep look at the individual daily life contexts, and their impact on physical activity and emotional experience while performing the regular daily activities. Gaining this insight is only possible by looking at the routine over several weeks. Mobile technology provides the means to gather real-time information in daily life for long periods of time [15]. Accelerometers and experience sampling have been successfully used in the past to evaluate the contexts of sedentary behaviour among older adults [16], in which most of the sedentary time was performed within the home environment and alone. Also, the influence of contexts on positive affect during physical activity has been evaluated using a similar method with the adult population in which it was reported that social activities might enhance positive emotions while performing physical activity [17]. However, none of the studies above mentioned looked at several weeks period within individual. Our study intends to extend the previous studies by investigating: (1) *how do daily activities relate to physical activity*, (2) *how do daily activities relate to the experience of pleasure,* and (3) *how do daily activities influence the relation between physical activity and pleasure in the daily lives of the older adults*. Combining the information gathered on the influence of daily activities on physical activity and experience of pleasure in daily life, we can further work on developing technology-ased interventions that will support older adults becoming more active through the promotion of pleasurable activities. Moreover, this research contributes to the understanding of emotions in the daily lives of the older population.

### Research Hypothesis

We establish that daily activities are defined by five categories: location (where the person is), activity (what the person is doing), social companion (with whom is the person doing the activity), emotional experience (how is the person feeling) and physical activity (amount of movement performed). Similar categorization is adopted in other studies relating daily context information to physical activity in daily life (e.g. [16, 17]). We have four hypotheses that are investigated in the current study.

- *H1. Social activities are more pleasurable than activities performed alone.* The social environment plays a clear role on the wellbeing of the population. Participation in social activities has constantly been associated with higher experience of general wellbeing [18] and positive emotions [19–22].
- *H2. Outdoors activities are more pleasurable than activities performed at home.* Previous research suggests that outdoor activities are associated with higher levels of positive emotions in the older population [19, 23, 24]. However, Gagliardi et al., when comparing German and Italian population, found a significant relation in the German population but not in the Italian, suggesting that there are cultural differences [19].
- *H3. Leisure activities are more pleasurable than basic activities of daily living.* Time spent in recreational/hobby activities is a predictor of daily mood independently on level of cognitive impairment [23]. Participation in leisure activities increases overall levels of positive emotions [20] and delays functional decline [25].
- *H4. There is no relation between physical activity and pleasure.* There is solid evidence for the benefits of exercise programs on mental health [26, 27]. However, the relation between physical activity and positive emotions is much less explored. There is some small evidence for relation between positive emotions and physical activity [28] but further work needs to be done to understand the mechanisms influencing this relation. We hypothesize that there is no relation because we choose an emotion, pleasure, that is not, *per se*, associated with high or low arousal emotions. Pleasure can be experienced with very relaxing activities but also with very exciting ones.

## Methods

### Participants and Setting

Ten community-dwelling volunteers were recruited from the area of Enschede, the Netherlands. Volunteers were invited for an interview in office, or at a location of their choice, in which the background, objectives and setting of the study were explained. Duration of the interviews was adjusted according to the technology affinity of each subject. Older adults included in the study reported being actively engaged in the community (e.g. performing volunteer work or integrated in associations) and did not have any limitations on activities of daily living, assessed with the Katz Index [29]. These inclusion criteria aimed to identify role models in the older population. When these criteria were met, the system composed of an accelerometer and a mobile phone was given to the subjects, together with a detailed manual explaining all the functionalities. The participants were encouraged to contact the research team in case of any doubt or problem with the technology. After 2 weeks, the researchers contacted the participants to guarantee that they were still engaged with the research. Approximately 1 month after the first interview, the participants were contacted to set up a new meeting to return the technology and finalize the study. The first five subjects participated in the study between October and November of 2014 and the other five in the same months of 2015. Written informed consent was obtained from all volunteers, and a small compensation was provided for participating in the study.

Table 1 summarizes the demographic and health related information of the subjects. Ten older adults (aged 65–83; *M* = 68.7, SD = 5.5, four males) participated in the study during approximately 30 days (range 24 to 38 days). Three participants were living alone at the time of the study and all participants had a computer and internet at home. Nine participants were considered robust and one was pre-frail. None of the participants had physical or cognitive functioning limitations. All participants had normal nutrition status according to the MNA; however, four participants were pre-obese and one was obese, on basis of the BMI.

**Table 1:** Basic characteristics of study participants

Characteristic	*N* = 10
*Age*	
Mean (SD)	68.7 (5.5)
range	65–83
*Male*	4
*Living alone*	3
*PC at home*	10
*Internet at home*	10
*BMI, mean (SD)*	25.2 (3.5)
Normal Weight	5
Pre-obese	4
Obese	1
*Frailty Level*	
Frail	0
Pre-frail (score)	1 (4)
Robust (range scores)	9 (0–3)
*Physical functioning, mean (SD)*	91.0 (8.3)
Limitations	0
No limitations (range scores)	10 (70–100)

### Measurements

#### Health status

Health condition was assessed on basis of frailty and in three specific domains: physical, cognitive and nutritional. This assessment follows the frailty screening used within the PERSSILAA project (http://www.perssilaa.eu). General frailty was assessed with the Groningen Frailty Indicator [30], in which a score higher than four indicates ‘frailty’. Physical limitations were assessed using the Katz Index [29] (score higher than four considered robust) and the physical functioning scale of the Short Form-36 Health Survey [31] (score higher than 60 considered robust).

#### Physical activity

Physical activity was measured throughout the day with the Activity Coach, a system composed of a hip-worn three-axial accelerometer and a smartphone application (for more information on the platform refer to [32]). This application has been used in several research projects in the rehabilitation and health promotion field in the past (e.g. [33–35]). The acceleration was quantified as Integral Module of Acceleration (IMA) per minute, with a_x_, a_y_ and a_z_ representing the accelerometer output in the three dimensions and T the time interval of integration, as presented in Equation (1):

$$ IMA(t)=\frac{1}{t}\left({\displaystyle {\int}_{t-T}^t\left|{a}_x\right|dt}+{\displaystyle {\int}_{t-T}^t\left|{a}_y\right|dt}+{\displaystyle {\int}_{t-T}^t\left|{a}_z\right|dt}\right). $$

For more information refer to [36]. The participants did not receive any physical activity goal or feedback during the measurement period.

#### Daily environments

The daily environments and pleasure experienced during the activities were assessed by experience sampling method [37] on the Activity Coach smartphone application. The participants were prompted approximately every hour from 8 AM to 8 PM with a question asking what they were doing at that moment. A set of common activities (e.g. preparing food, eating, resting, and playing with children) was shown on the screen as well as the option to manually input an additional activity. After reporting the activity, the subjects were asked about the location where the activity took place (home, workplace, somewhere outside or somewhere inside) and the social companion while performing that activity (e.g. alone, spouse, and friends). The name of the activity was continuously displayed so that the participants had a reference on the activity they were reporting on.

#### Pleasure

Pleasure is the outcome variable of this study and it was assessed on the smartphone. Pleasure is an important concept within health promotion research as, according to the Self-Determination Theory [13], previous experience of pleasure while performing an activity is an intrinsic motivator to repeat that activity. Therefore, a focus on pleasurable activities is expected to enhance healthy behaviours. Pleasure, being a composite variable, knows different extents of arousal or activation, from calmness to alert. However, as a first explorative study and do not wanting to increase the demand from the participants, we have chosen pleasure as an operationalization of the positive semi-axis of the valence dimension of emotions. In other words, participants were asked about their general experience of pleasure, not looking at whether that experience was accompanied by an experience of low activation (e.g. calmness) or high activation (e.g. excitement). For more information about the circumplex model of affect refer to the work of Russell over the past 30 years. After reporting on the activity currently being performed, location and social companion, the respondents rated on a visual analogue scale, ranging from 0 (not at all) to 10 (totally), how pleasurable that activity was to perform.

Participants were measured for a total of 320 days. In total, 2301 experience sampling points (ES-points) were collected with the number of points per subject ranging from 186 to 318. When looking at the whole sample, approximately three thirds of the activities were performed at home (59–88%), more than half of the activities were performed alone (30–96%) and the proportion of activities reported as bADL and leisure was approximately 50% (33–58%). Table 2 summarizes the characteristics of the study and frequency of answers. Outcome variable pleasure did not show severe deviations from normality for the whole sample neither within subject.

**Table 2:** Study time characteristics of all participants and range per subject

Parameter	All participants	Range per participant
*Measurement days*	320	24–38
*ES-events*	2301	186–318
*Pleasure*		
mean	7.31	5.75–8.73
SD	1.58	0.48–2.14
*Location*		
% activities at home	72.5	58.5–87.9
*Social Companion*		
% activities alone	56.9	30–96
*Type of Activity*		
% bADL activities	45.5	32.9–57.9

### Data analysis

#### Pre-processing

The outcome variable pleasure was transformed to provide an indication of variability. Considering that some subjects only use part of the scale (e.g. 7–9 points) and others used the full scale, using the raw values, an increase of 0.5 points in pleasure would represent a significant change for some subjects, but not for others. Therefore, we normalized the values of pleasure within subject so that 0 and 100 correspond to the minimal and maximal value of pleasure, respectively. In this case, 50 (per cent) corresponds to the median value of pleasure reported by each subject. The tests for the distribution of the transformed pleasure suggest normality in terms of skewness (−0.9) and kurtosis (2.49).

To calculate the amount of physical activity performed during an activity, we established, for each experience sampling event (ES-event), a 10-min time window centred in the moment of answering the questionnaire on the smartphone. The total IMA performed during this period was considered. It is known from previous technical trials that sensors can provide abnormal high values of IMA, for example, due to bumps. Therefore, outliers of IMA values were filtered, resulting in a pre-analysis filtering of 2% of the data points. Due to the high positive skewness of the raw values of IMA, we applied the cubic root and the data distributions suggested normality (skewness 0.08, kurtosis −1.1). Finally, outliers were removed resulting in a final sample of 2219 ES-events.

Types of activities, locations and social companions were categorized in dichotomous variables. Activities related to self-care, eating, performing households and commuting were considered as basic activities of daily living (bADL) as these are activities that each individual is, somehow, obliged to do. All other activities were classified as ‘leisure’ activities. This distinction was not performed on basis of the intensity of physical activity associated with each daily activity, but, instead, on basis of the assumption of the motivation why the individual performed each activity. While the activities defined as bADL correspond the activities that the person has to do (for surviving or for being able to perform other activities, as in commuting), the activities considered as leisure incorporate all the non-mandatory activities, such as going out or relaxation. Location was dichotomized as ‘at home’ and ‘not at home’. In terms of social companion, each event was classified as being performed ‘alone’ or ‘not alone’.

#### General mechanisms predictors of physical activity in daily life

Multilevel regression analysis were performed using SPSS version 22 with repeated measurements nested within subjects. Fixed- and random effects were calculated with type of activity, location and social companion as predictors.

#### General mechanisms predictors of pleasure in daily life

Multilevel regression analysis was also performed in this case with repeated measurements nested within subjects. Model 0 was the null model (without predictors) revealing how much variance in pleasure was associated with subject differences. In model 1 we added the main effects of the daily environments (i.e. type of activity, location and social companion). Model 2 included physical activity as a predictor of pleasure. Based on graphical visualization of variability of pleasure, we denoted that the day of the week, particularly Monday’s, seemed to have an influence on pleasure. Therefore, in model 2 we added ‘Monday’ as predictor of pleasure. Model 3 included interaction between physical activity and each property of daily environments. Finally, Model 4 included the subject-level predictors (age, gender, frailty indicator, physical functioning and body mass index (BMI)).

#### Individual mechanisms analysis

We have tested the possibility of including random slopes but the models could not be reliably estimated by SPSS, leading to problems of convergence, most likely due to the small sample size (*N* = 10). Therefore, we decided to run models only with a random intercept. Post hoc random variability was investigated by performing linear regression models (10 times *N* = 1).

## Results

### General mechanisms predictors of physical activity in daily life

Type of activity, location and social companion while performing an activity significantly predicted physical activity in our sample (*p* < 0.001). Leisure activities required less physical activity than bADL (*b* = −3.23, *t* = −11.92). Regarding the location, activities performed outdoors were associated with higher levels of physical activity (*b* = −3.28, *t* = 10.14). Social activities required, in general, less physical activity than activities performed alone (*b* = −1.96, *t* = −6.43). There was significant variance of intercept across models suggesting variability between subjects. Table 3 provides the results of this analysis. Random slopes analysis were almost significant for social companion and type of activity, suggesting significant different effect between subjects.

**Table 3:** Predictors of physical activity in the daily life of older adults living independently

	Model 0	Model 1		
Predictors	*b*	*b*	*t*	*p*
Repeated Measurement-level				
Intercept	21.561	23.306		
Type of activity		−3.230	−11.919	<0.001
Location		3.275	10.139	<0.001
Social companion		−1.957	−6.428	<0.001
Variance components				
Between subjects	3.516^*^	3.460		0.036
Model fit statistics				
-2Log Likelihood	14738.977	14499.516		

Predictors level 1: type of activity (leisure = 1), location (not at home = 1) and social companion (with someone else = 1)

*p < 0.05

### General mechanisms predictors of pleasure in daily life

In the following analysis we used the normalized values of pleasure within subject with zero corresponding to the minimum value of pleasure indicated by the subject, 100 to the maximum and 50 to the median value. An intra-class correlation of 0.41 provided strong evidence to nest repeated measurements within subjects, meaning that 41% of the variation of pleasure between measurements could be explained by subject differences. Table 4 provides the results of multilevel models 0 to 4.

**Table 4:** Predictors of pleasure in the daily lives of older adults living independently

*N* = 2219	Model 0	Model 1	Model 2	Model 3	Model 4
Predictors	*b*	*b*	*b*	*b*	*b*
Repeated Measurement-level					
Intercept	−1.842	−10.572	−8.811	−6.535	−10.872
Type of activity		9.875***	9.360***		
Location		2.050*	2.475**		2.057*
Social companion		6.259***	5.890***		5.781***
Weekday			−2.135*	−2.136*	−2.153*
Physical Activity (IMA)			−0.088*		
*Interactions*					
IMA * Type of activity				0.312***	0.300***
IMA * Location				−0.073	
IMA * Social companion				0.014	
Subject-level					
Gender					2.908
Age					−0.142
BMI					0.421
GFI					2.053^¥^
SF-36					−0.245
Variance components					
Between subjects	0.066	0.170	0.259	0.237	0.590
Model fit statistics					
-2Log Likelihood	19054.143	18811.197	18800.103	18777.224	18772.324

Predictors level 1: type of activity (leisure = 1), location (not at home = 1), social companion (with someone else = 1), weekday (Monday = 1) and physical activity (IMA). Predictors level 2: gender (male = 1), age (years), BMI, result of GFI and results of SF-36

*IMA* Integral of Modules of Acceleration

^¥^ *p* < 0.1, *p < 0.05, **p < 0.01, ***p < 0.001

**Table 5:** Results of the regression analysis for each participant in the study

	*Participant*									
	1	2	3	4	5	6	7	8	9	10
Predictors	*b*	*b*	*b*	*b*	*b*	*b*	*b*	*b*	*b*	*b*
Physical Activity (IMA)	−0.123	−0.637	−0.265	−0.450**	0.017	−0.194	0.063	−0.421*	−0.063	−0.099
Type of activity	2.546	10.317**	0.325	−7.672	5.727	3.230	7.515*	9.998*	11.747**	6.508**
Location	2.139	−3.150	8.130**	5.273	−0.279	−1.692	−0.861	4.202	1.671	0.632
Social companion	3.362*	14.913***	0.387	15.651***	1.465	−0.050	6.365**	15.111***	−1.198	3.450
Weekday	0.250	−12.398**	0.881	−1.507	−3.649	1.991	−4.809	−3.530	0.400	0.357
Physical Activity * Type of activity	0.086	0.900	0.309	0.897***	0.342	0.311	−0.115	0.504	0.024	0.007
R-squared	.081	0.384	0.082	0.194	0.089	0.042	0.102	0.350	0.098	0.150

Predictors: physical activity (IMA), type of activity (leisure = 1), location (not at home = 1), social companion (accompanied = 1) and weekday (Monday = 1)

*p < 0.05, **p < 0.01, ***p < 0.001

#### H1. Social activities are more pleasurable than activities performed alone

Social companion (alone vs. with someone else) is a strong predictor of the experience of pleasure while performing an activity, despite the other predictors. Activities performed with someone else provided 6% more pleasure than activities performed alone (*p* < 0.001, Models 1, 2 and 4), confirming our initial hypothesis.

#### H2. Outdoors activities are more pleasurable than activities performed at home

Outdoors activities resulted in an increase of pleasure of 2% above the median value when compared to activities performed at home (*p* < 0.05, Models 1 and 4, *p* < 0.01 Model 2), also when adding all other predictors, confirming our hypothesis.

#### H3. Leisure activities are more pleasurable than basic activities of daily living

Type of activity (leisure vs. bADL) was found to be the strongest predictor of pleasure. Performing a leisure activity, results in an increase of 10% of pleasure above the median when compared to bADL (Models 1 and 2, *p* < 0.001), confirming the hypothesis.

#### H4. There is no relation between physical activity and pleasure

**Fig. 1:**
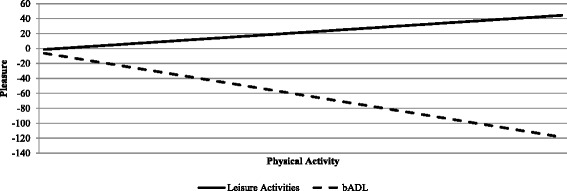
Relation between physical activity (IMA) and deviation from the median value of pleasure when performing leisure activities (*solid line*) and bADL (*dashed line*). When looking at the full sample of activities, more physical activity relates to less experience of pleasure. However, when looking only at the subsample of leisure activities, more physical activity is associated with more pleasure while performing the activity

Contrarily to what hypothesized, amount of physical activity while performing an activity was a weak, but statistically significant, predictor of pleasure, with more physical activity resulting in 2% less experience of pleasure (Model 2, *p* < 0.05). When looking at the interaction effects between physical activity and properties of daily living, only the interaction between type of activity and physical activity was a significant predictor of pleasure (*p* < 0.001, Model 3). While engaged in leisure activities more physical activity results in higher levels of pleasure while in bADL, more physical activity results in less experience of pleasure (see Fig. 1).

### Post-hoc analysis

None of the subject level characteristics was a significant predictor of pleasure at *p* < 0.05. Based on post-hoc analysis, we included Monday as a predictor of pleasure. Activities performed on Monday’s resulted on less pleasure than activities performed on the remaining weekdays (*p* < 0.01, Models 2–4). This effect remains significant even when adding other predictors and interactions.

### Individual mechanisms

As the reduced sample size did not allow us to look at random effects, we performed individual analyses with linear regression models. The predictors are the same as in the Model 3 of the multilevel analysis. Table 5 provides the results of this analysis, with the strength of the predictors differing strongly among participants. Whereas the pleasure of some subjects is significantly predicted by one or two properties of daily environments (e.g. participant 2 and 4), the experience of pleasure of other participants does not appear to be influenced by daily environments (e.g. participant 5 and 6). For three out of the ten participants, activities performed accompanied represented approximately 15% more pleasure than their personal median value of pleasure, social companion being the most relevant determinant of daily environments in pleasure. We can see that for both the full sample and individual participants, performing an activity with someone else predicts higher pleasure than performing the same activity alone.

**Fig. 2:**
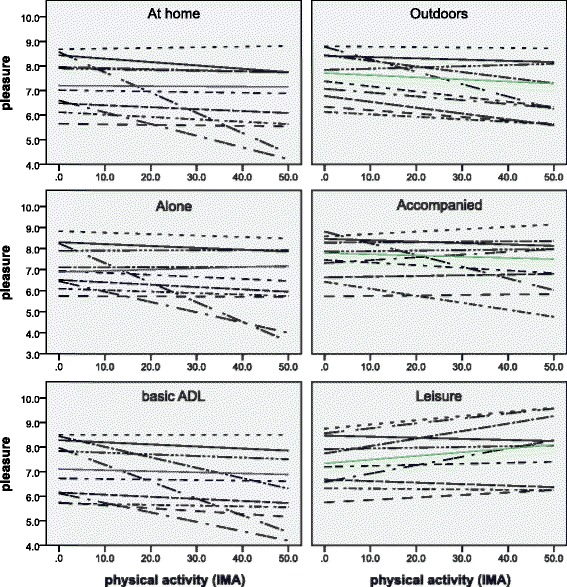
Impact of daily activities and their contexts on the relation between physical activity and pleasure. Individual (*dashed lines*) and group trends (*blue and green lines*) on the relation between physical activity and pleasure categorized according to location (*top*), companion (*middle*) and type of activity (*bottom*)

Figure 2 shows the relation between physical activity and pleasure for each subject and the average for the overall sample population considering the location, social companion and type of activity performed. Also visually individual differences are clear with for example, opposite slope signs when looking at the outdoors context. In this case, the original values of pleasure are shown (i.e. not the normalized values) in order to provide a loyal representation of the individual answers. Furthermore, this representation provides a clearer visualization considering that the participants use different parts of the pleasure scale, as already mentioned, and we want to show the variability within individual.

## Discussion

The objectives of this study were to perform an exploratory investigation on (1) how daily activities relate to physical activity, (2) how daily activities relate to the experience of pleasure, and (3) to which extent these daily activities explain the interaction between pleasure and physical activity, in the daily lives of community-dwelling older adults. Data was collected with an accelerometer and experience sampling on a smartphone. No feedback was provided to the participants during the study. The results of our study confirm the first three hypothesis which stated that social activities, outdoor activities and leisure activities are more pleasurable than activities performed alone, indoor activities and basic activities of daily living, respectively. However, the last hypothesis, stating that there is no relation between physical activity and pleasure, is partially rejected. This result suggest that the type of daily activity as a moderator effect in the relation between physical activity and pleasure.

This research was designed to gather information for the design of strategies to promote physical activity through recommendation of pleasurable activities. Preliminary recommendations for technology development can be drawn from the presented study.

First, our results support the idea of tailoring interventions when promoting pleasurable activities. Although general effects can be taken from the full sample, looking at the results of the within-individual analysis, we see that, as expected, the predictors of pleasure are highly personal. By gathering data over approximately 30 consecutive days (ranging from 24 to 38, according to the availability of each individual), we can conclude that there are individual differences. Taking into account the market growth of mobile devices to monitor physical activity, also outside the scientific domain, in the future, individual differences might be detected automatically using data mining techniques. Interventions can thus be tailored to the preferences and needs of the individuals, even in the cases that the preferences change over time.

Second, older adults spend most of their time at home and alone. This fact is certainly not surprising, but the proportions are, by the fact that our sample was relatively healthy, and active in the community, representing, what we named as, the role models. The study took place during Winter time, and therefore, people are more likely to spend time at home. However, it is still remarkable that, for example, in one of the subjects, 88% of the activities reported during 1 month took place at home and 96% alone. The World Health Organization emphasizes the importance of being engaged in the community and environments for a healthy lifestyle [3]. Interventions that stimulate social inclusion of older adults are highly recommended as well as interventions that coach the individual to go outdoors, as the home setting is where the older adults spend most of their sedentary time [16].

Third, motivation of physical activity *by proxy* is recommended based on our results, expanding the results from [28]. By motivation by proxy, a coaching strategy that motivates people to engage in outdoor- or social activities, increasing physical activity indirectly. For example, instead of recommend an individual to go for a walk, one can inform about a new exhibition in the local museum. By going to this exhibition, the individual needs to move. Combining the results from objective 1 and 2, we see that promotion of outdoors activities are the most valuable considering that these activities result both in higher experience of pleasure and more physical activity. Promotion of leisure activities is also highly recommended, as the experience of pleasure increases with physical activity when individuals are engaged in leisure activities, but not in bADL. *Post-hoc* analysis suggests that this effect in bADL is due to household activities. It is known that household activities are a source of physical activity in the daily living [38], however, our study suggests that it is not the most pleasurable one, and therefore, not likely to be a good motivator to perform physical activity.

Fourth, we can see that 30 days of measurement, with approximately six events per day, generates enough data to analyse the influence of daily environments on the experience of pleasure. However, this is a very demanding procedure which should be reduced in the future. Future research could investigate whether it is possible to obtain the same degree of information with a shorter study. Participants of the study reported that answering questions every hour for 1 month is an annoying task. However, identifying what is pleasurable for each individual without becoming cumbersome remains challenging. New technological developments such as emotion recognition tools (either using facial expression recognition or bio sensing) might provide the means for less obtrusive research in this area. Automatic assessments of emotions assume even higher importance when seeing that the interpretation of the feeling pleasure and quantification in a scale is highly personal. While some subjects made use of the full scale (0 to 10) others limited themselves to a short range. This might have to do with personality or with other factors. This means that looking at the exact place of the VAS chosen is not a good measure. Instead, in our data analysis we normalized the values to correspond to a deviance from the median. Rocke et al. reported that older adults report low variability rates of positive affect when compared to younger adults [39]. The use of the hip-worn accelerometer can also become obtrusive. This is likely to be overcome rapidly with the consumer oriented lifestyle devices to promote physical activity becoming smaller, and being used by more people every year. Objective monitoring of physical activity of the older population might be a promising addition to conventional questionnaires, as there is evidence that the objective measurement provides more reliable information [40].

To the best of our knowledge, our exploratory study is innovative for the variety of data gathered, and the combination of methods used to gather information during the course of 1 month among the older population. From each participant, we obtained health related information, lifestyle behaviours, emotional- and context-information for a period of approximately 30 days. Furthermore, we made use of three distinct data acquisition methods: conventional standardized questionnaires, on-body sensing and experience sampling. These factors combined provide very valuable knowledge, because, contrarily to most of the studies developed in the past, our data was acquired in real-time, in the daily life of the participants, instead of using a questionnaire that asks previous experiences. We believe that only in this way one can get a reliable view of the daily behaviours. Further research should be performed by analysing more distinct categories of the properties of daily environments, instead of dichotomous variables. For example, in terms of social companion, one could look at how the experience of pleasure and physical activity are influenced by the fact that an activity is performed with the partner, relatives or even specific friends. Future research could also look separately at routine and non-routine activities. Bouisson & Swendsen suggest that breaks in the routine improve the wellbeing of the older adults, even the ones who claim that they prefer routine [41]. Finally, we encourage researchers to verify if tailored interventions for promotion of physical activity based on the preferences and enjoyment of the individuals do increase adherence, as suggested by the Self-Determination Theory [13].

Our study has limitations. First, although the subjects were told to adapt the measurement period to their own routine, the battery of the phone and the accelerometer limited the measurement period to a maximum of 12 h. Considering that mostly the subjects would start measuring in the morning, the evening period was not considered in our studies. Assuming that in the evening period, people are more likely to do relaxation activities within the home environment, this means that people spend more time at home than reported. Secondly, the visual analogue scale was experienced as being difficult to use, as the interpretation of the scale differed per participant, as well as where to locate the finger in the scale. For future studies we suggest the use of Likert scales instead of visual analogue scales. Still regarding the outcome variable, in this study we were only interested in investigating the experience of positive emotions. To avoid over complicate the study, we have only looked at the valence dimension (positive axis “Pleasant”) and did look at different experiences of the arousal, or activation, dimension. We recommend researchers to look at the several discrete emotions corresponding to different arousals, from deactivation to activation, following the circumplex models of emotion [42, 43]. Regarding the data analysis there are two points for discussion. First, we reduced the granularity of the data grouping variables in dichotomous variables. Although this grouping provides already very interesting results, further analysis should be performed with the original categories. Second, in the calculation of the physical activity related to a daily activity, we considered a time window of 10 min centred in the moment of answering the question. This is likely to be arguable, however, our sensitivity analysis with several lengths and time shifts did not show any significant difference. Finally, there is clear the issue of the small sample size. However, as stated in our objectives, we were aiming at getting deep insight in within person analysis, and that objective was met. Further studies should be performed with a larger sample and look at possible predictors of pleasure and physical activity, such as depression or years after retirement.

## Conclusions

Our exploratory study suggests that daily activities and their environments do have an impact on the experience of pleasure, physical activity, and relation between physical activity and pleasure, with significant differences between subjects. We also see that older adults are willing to use wearables for periods of 1 month. . Based on this exploratory study, the use of sensors and experience sampling seems a promising addition to the conventional questionnaires to investigate the relation between physical activity and positive emotions and the mediator effect of the daily activities.
